# Inherent Safety Assessment of Industrial-Scale Production of Chitosan Microbeads Modified with TiO_2_ Nanoparticles

**DOI:** 10.3390/biom11040568

**Published:** 2021-04-13

**Authors:** Samir Meramo-Hurtado, Nicolas Ceballos-Arrieta, Jose Cortes-Caballero, Jeffrey Leon-Pulido, Arturo Gonzalez-Quiroga, Ángel Dario Gonzalez-Delgado

**Affiliations:** 1 Chemical Engineering Program, Universidad EAN, Bogotá 110221, Colombia; jleonp@universidadean.edu.co; 2 Chemical Engineering Program, Universidad de Cartagena, Bolivar 24120, Colombia; nceballosa@unicartagena.edu.co (N.C.-A.); jcortesc@unicartagena.edu.co (J.C.-C.); agonzalezd1@unicartagena.edu.co (Á.D.G.-D.); 3 UREMA Research Unit, Mechanical Engineering Department, Universidad del Norte, Barranquilla 25138, Colombia; arturoq@uninorte.edu.co

**Keywords:** inherent safety index, aspen plus®, chitosan microbeads, TiO_2_ nanoparticles

## Abstract

In this study, the inherent safety analysis of large-scale production of chitosan microbeads modified with TiO_2_ nanoparticles was developed using the Inherent Safety Index (ISI) methodology. This topology was structured based on two main stages: (i) Green-based synthesis of TiO_2_ nanoparticles based on lemongrass oil extraction and titanium isopropoxide (TTIP) hydrolysis, and (ii) Chitosan gelation and modification with nanoparticles. Stage (i) is divided into two subprocesses for accomplishing TiO_2_ synthesis, lemongrass oil extraction and TiO_2_ production. The plant was designed to produce 2033 t/year of chitosan microbeads, taking crude chitosan, lemongrass, and TTIP as the primary raw materials. The process was evaluated through the ISI methodology to identify improvement opportunity areas based on a diagnosis of process risks. This work used industrial-scale process inventory data of the analyzed production process from mass and energy balances and the process operating conditions. The ISI method comprises the Chemical Inherent Safety Index (CSI) and Process Inherent Safety Index (PSI) to assess a whole chemical process from a holistic perspective, and for this process, it reflected a global score of 28. Specifically, CSI and PSI delivered scores of 16 and 12, respectively. The analysis showed that the most significant risks are related to TTIP handling and its physical-chemical properties due to its toxicity and flammability. Insights about this process′s safety performance were obtained, indicating higher risks than those from recommended standards.

## 1. Introduction

Industrial production and human development dynamics have led to resource consumption rates (mostly non-renewable) that exceed the natural provision capacity [1]. Productivity activities based on the production of goods and services have generated impacts on the environment, affecting abiotic and biotic ecosystems around the planet [2]. In this sense, many processes of critical economic sectors such as chemical, petrochemical, and transportation industries, among others, have affected water sources by discharges to surface waters with high hydrocarbons and heavy metals concentrations [3]. One of the industries that present issues related to waste management and the potential impacts on the environment is the shrimp production sector [4]. In Colombia, shrimp production is estimated at 2400 t/year, of which 20% is discarded as waste. The chemical composition of shrimp exoskeleton (30–40% *w*/*w* chitin) makes this substance suitable to produce chitosan [5]. Chitosan is a substance used as a final product (for the cosmetic and food industries) or raw material to produce bio-adsorbents with wastewater treatment applications [6].

Currently, nano-technology represents an opportunity to connect research and product development applied to compounds and processes that have positive impacts both technically and ecologically [7]. It is known that TiO_2_ is used in photocatalytic systems to suppress organic pollutants in water [8]. Studying the production of novel biotechnology-based adsorbents becomes relevant, including their modification with nanomaterials. The above involves generating added value to clean production and obtaining more specific absorption characteristics and environmentally friendly processes under Green Chemistry principles [9]. There is a positive perspective about advancing novel technologies based on biomaterials, nano-technology, and sustainable development principles that permit us to meet needs, starting from the aim to decrease environmental impacts, increase profits, and enhance social benefits [10].

In this study, the production of chitosan microbeads modified with TiO_2_ nanoparticles is analyzed due to the potential use of this product to remove contaminants from water (for example, hydrocarbons), for adsorption, and also for photocatalysis [11]. An important aspect that must be considered in the design of any physicochemical process is determining the inherent risk levels. This factor implies developing risk assessments considering human resources (operators, supervisors, management employees), process operating conditions, and chemical compounds handled throughout the plant. Therefore, safety analysis is an invaluable tool for designers and decision-makers [12]. This research presents an evaluation of the inherent safety of the production process of chitosan microbeads modified with TiO_2_ nanoparticles through the Inherent Safety Index and computer-aided process engineering. It should be noted that the authors presented a previous work studying environmental and exergy aspects of this process topology using computer-aided process engineering [13].

Various methods are reported in the literature to perform chemical process safety assessments. In this sense, methods such as Dow Fire and Explosion Index (Dow F&EI), Prototype Index of Inherent Safety (PIIS), and Inherent Safety Index are some of the most widely used safety indexes in industrial processes, each one with their respective measurement procedures and methods (and with their advantages and disadvantages) [14]. In this work, the safety evaluation was developed using the traditional Inherent Safety Index (ISI) method developed by Heikkila [15]. This methodology has the advantage of providing insights concerning associated inherent risks of a chemical plant measured from conceptual design information (such as mass and energy balance, temperature, and pressure, among others). The ISI analysis includes evaluating the chemical risks of the substances involved in the process and its equipment and operating structure. These risks are calculated by using a mixed approach, including quantitative and qualitative overviews [16].

Different authors have proposed various modifications and extensions of the ISI method depending on the scope and approach. Koller [17] proposed evaluating the early impact of safety, health, and environment during the development of processes based on 11 categories of impacts (such as; mobility, reaction, irritation, among others). Process safety has been used to assess associated risks of oil palm production under the Hazard Identification and Risk Assessment (HIRA) method [18]. Kham and Amyotte [19] proposed a novel index called the Integrated Inherent Safety Index (I2SI), which incorporates evaluating the process’s life cycle with an economic evaluation and identifying the potential dangers of the operation. In this sense, Rathnayaka et al. [20] developed the Risk-based Inherent Safety Index (RISI), which incorporates both the reduction of consequences and the probability of an accident occurring through inherently safer application design principles throughout the life cycle of process design. Process safety analysis has also served to study the risks and hazards of topologies for shrimp exoskeleton valorization using the Numerical Descriptive Inherent Safety Technique (NuDIST) [21].

In summary, this work presents a safety evaluation of large-scale topology production to synthesize chitosan microbeads modified with TiO_2_ nanoparticles under the inherent safety index methodology, which provides a holistic perspective of how this process would behave during industrial operation. This topology is an emerging technology under development and research at its early or conceptual design stage. Therefore, this paper presents new insights into the continuous progress of this type of bio- and nano-technologies considering future development from a process safety viewpoint.

## 2. Materials and Methods

### 2.1. Process Description

The process was designed for a production capacity of 2032.55 t/year of chitosan microbeads modified with TiO_2_ nanoparticles. TiO_2_ nanoparticles are produced via green chemistry using lemongrass extracts and TTIP hydrolysis [13]. The global process consists of two main stages. Green synthesis of TiO_2_ nanoparticles comprises two subprocesses: (a) Extraction of lemongrass oil, and (b) synthesis of TiO_2_ nanoparticles. The second stage is the production of modified chitosan microbeads. For subprocess (a) in stage 1, lemongrass is initially pretreated through cleaning and washing operations to remove cellulosic material. Subsequently, the process stream is sent to a dryer (to reduce humidity) that elevates temperature to 95 °C and then cooled to ambient temperature (28 °C). The outlet stream is directed to the grinding unit to reduce the particle size of pretreated lemongrass. The oil extraction is carried out through a solid–liquid separation through an infusion mixture with water for subsequent separation by decantation. The herb oil is mainly composed of myrcene, neral, geranial, citral, and nerol, among other phytochemicals, with a total composition of 1.10% wt. [22]. Finally, the mainstream with diluted oil is sent to an evaporation stage to reduce the water content. A stream of 5714.75 t/year of phytochemicals was obtained with an oil content of 4.2% w/w from the feeding of 33,000 t/year of lemongrass. A previous investigation [23] from our group assessed the environmental and ecological impacts of green-based synthesis of TiO_2_ nanoparticles using a configuration similar to that described in this work.

Concerning subprocess (b) in the first stage of the process, the TiO_2_ nanoparticles are formed using TTIP (5724.16 t/year) as the primary raw material through hydrolysis. The lemongrass extract stream is sent to the reactor as a surfactant medium to guarantee the nano-size. Equation (1) represents the hydrolysis reaction for the formation of TiO_2_ nanoparticles from TTIP [8].

Ti(OC_3_H_7_)_4_ + 2H_2_O → TiO_2_ + 4C_3_H_7_OH (1)

In a stage before the hydrolysis reaction, the pure TTIP is sent to a mixing tank to create a mixture with water. The extracted oil and reagent flows enter the hydrolysis reactor after a rapid water-TTIP mixture tank. A yield of 0.93 mol of TTIP was obtained to produce 1.00 mol of TiO_2_ based on laboratory-scale experiments [23]. The hydrolysis reaction occurred at 28 °C and 1 atm. The main by-product of the reaction is propanol, which is removed to avoid nanoparticle contamination. The reactor outlet streams are sent to a purification stage, which employs drying units and centrifuges. Finally, calcination is carried out to obtain completely dry TiO_2_ nanoparticles, which are sent to the next processing unit [24].

**Figure 1:**
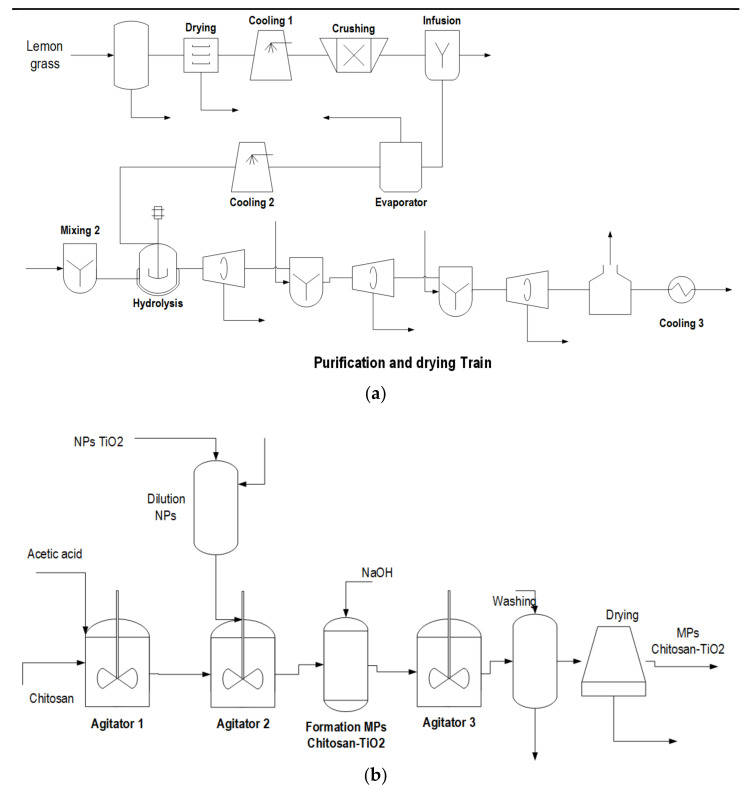
Process diagram of chitosan microbeads modified with TiO_2_ nanoparticles production. (**a**) Process stages (1) and (2). (**b**) Process stage (3).

The second stage of the process begins by preparing chitosan and acetic acid (diluted at 2% *w*/*v*) solution for gelation. In parallel, the TiO_2_ nanoparticles are diluted to the same concentration as acetic acid with a 1:1 ratio for the chitosan mass flow. Subsequently, the diluted solutions are mixed in a mixing tank system. The precipitation of the modified microbeads is achieved under alkaline conditions, so adding a NaOH stream (5 M) is needed to meet the required conditions. It is essential to mention that product formation in this system is merely physical, so it is expected not to present chemical reactions. The mainstream leaving the mixing tank is sent to a separation unit for pH neutralization and water content removal since it needs to be dried. This separation system consists of stirring, washing, and drying equipment, obtaining a total product flow of 2242.04 t/year. Figure 1 shows the diagram of the production process of chitosan microbeads modified with TiO_2_ nanoparticles.

### 2.2. Process Safety Assessment

Risk assessment has become a vital task to design and analyze new chemical processes under the sustainable design approach [25]. This study assesses process safety issues using the inherent safety index methodology of scaled-up production of chitosan microbeads modified with TiO_2_ nanoparticles. The estimation of ISI counts the inherent risks allied with operational aspects and hazardous properties. The examination of chemical and biochemical processes at the conceptual-design stage is one of the most valuable features of the inherent safety index method from a process system engineering perspective. Other methodologies might require more detailed data that is not always available in early engineering designs [26].

Many parameters are involved in calculating the process′s global risk index through the Inherent Safety Index (ISI), which relates to the substances′ chemical and process risks contributions, according to Equation (2).

ISI = CSI + PSI(2)

CSI is the process chemical risk index, while PSI is the process risk index. The CSI index considers all chemical substance properties such as reactivity, explosivity, toxicity, and others. CSI is calculated according to Equation (3).

CSI = I_RM, MAX_ + I_RS, MAX_ + I_INT,MAX_ + (I_FL_ + I_EX_ + I_TOX_) + I_COR_ (3)

I_RM,MAX_ is the main chemical reaction subscript, I_RS,MAX_ is the secondary chemical reaction subscript, I_INT, MAX_ is the chemical interaction subscript, (I_FL_ + I_EX_ + I_TOX_) is the maximum sum of subscripts for hazardous substances, and I_COR_ is the subscript for corrosion. The ISI method assumes that the worst possible situation can occur; hence, most of the sub-indexes’ scores are taken considering the properties/variables maximum. As the method involved assigning specific scores of indexes based on their relative performance, reference scores, and ranges are given according to experience, indirect methods, or scientific literature [27]. Table 1 shows the subscripts and reference ranges to assign scores for CSI sub-indexes. Most of the needed data for estimating CSI sub-indexes can be found in the literature or the reported safety data sheets for substances based on the Occupational Safety and Health Administration (OSHA) guidelines. Equation (4) shows the subscripts required for the estimation of PSI.

PSI= I_I_ + I_T, MAX_ + I_P, MAX_ + I_EQ_ + I_ST,MAX_ (4)

I_I_ is the subscript for inventory, I_T,MAX_ is the subscript for maximum process temperature, I_P,MAX_ is the subscript for maximum process pressure, I_EQ,MAX_ is the subscript for equipment safety, and I_ST,MAX_ is the subscript for safe process structure. Table 2 shows the subscripts related to the calculation of risks per process.

## 3. Results

### 3.1. Material Characteristics

Chitosan microbead modified with TiO_2_ nanoparticles is a new composite material whose primary use is for application as a bio-adsorbent in water treatment plants to remove pollutants like polycyclic aromatic hydrocarbons and heavy metals. This material features the formation of chitosan-TiO_2_ with good crystallinity, thermal stability, and paramagnetic response due to the presence of TiO_2_ nanoparticles. We found that this material can provide outstanding efficiencies in adsorption processes, with up to 89% for removing polycyclic aromatic hydrocarbons. The presence of TiO_2_ might involve an enhanced photocatalytic process that boosts pollutants removal [28]. The ionic cross-connecting technique makes available synthesizing modified microbeads with semispherical shapes. This material showed large pore sizes and surface area. These outcomes result from the nanoparticles′ contribution, which enhanced porosity formation and enlarged surface area.

### 3.2. Safety Analysis Results

The simulation of the production process of chitosan microbeads modified with TiO_2_ nanoparticles was carried out through Aspen Plus ^®^. Process simulation data allowed developing the process safety analysis using the Inherent Safety Index. According to the methodology proposed in the ISI method, an evaluation of chemical properties and the process structure must be carried out. The primary chemical reaction is TTIP hydrolysis to form TiO_2_ (see Equation (1)). As the lemongrass oil extraction and microbead formation units are physical systems, no side reactions were considered for this process. The score assignment of chemical reactions (I_RM,MAX_ and I_RS,MAX_) is made considering the heats of reactions of an analyzed system, according to Equation (5).

(5) $$\Delta H_{R}=\sum H_{\mathrm{fprod}}-\sum H_{\mathrm{freac}}$$

∆H_R_ is the heat of reaction, $\sum H_{\mathrm{fprod}}$ is the enthalpy of formation of products, and $\sum H_{\mathrm{freac}}$ is the enthalpy of formation of reactants. According to these calculations, the weight for the subscripts of chemical reactions is calculated based on the following criteria; extremely exothermic (≥−3000 J/g), strongly exothermic (<−3000 J/g), moderately exothermic (<−1200 J/g), slightly exothermic (<−600 J/g), thermally neutral (≤−200 J/g), or endothermic. Table 3 shows the heat of reactions and the score assigned to the main chemical reaction subscript.

According to the results reported in Table 3, I_RM_ obtained the maximum score (I_RM_ = 4) since it is a highly exothermic reaction according to the heat of reaction. Since it was considered that there are no secondary reactions, I_RS_ = 0 is assigned.

Chemical interactions and hazardous substance sub-indexes are related to the substances′ characteristics and properties involved in the process. Therefore, those that potentially represent risks in the operation of this plant were identified. Substances like Ethanol (EtOH), propanol (PrOH), TTIP, acetic acid (AA), and NaOH were identified as the riskiest components in the production process of chitosan microbeads modified with TiO_2_ nanoparticles. Some of the toxicological and reactivity properties of hazardous substances are shown in Table 4. Chemical interaction considers unwanted reactions of process substances with materials in the plant area; the substance that may have more significant chemical interactions than the rest is rated. For the case of the evaluated system, the substance with the highest chemical interaction is TTIP. For this case, a score I_INT_ = 3 is assigned because its main risk is related to the formation of flammable mixtures with air. This score implies that a fleeting of this substance in the reactor (or oxygen entry) can generate highly flammable mixtures.

**Figure 2:**
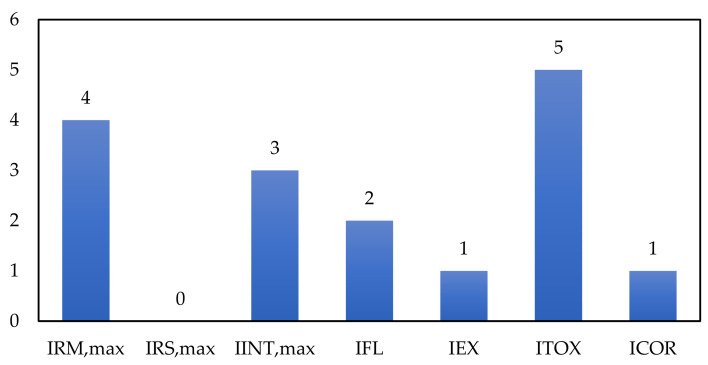
Contribution of CSI sub-indexes.

The dangerous substances sub-index is the sum of toxicity, flammability, and explosiveness parameters of compounds (see Table 4). The compound with the highest score from the sum of these indices is selected. In this sense, according to Table 4, TTIP is the compound with the highest sum of dangerous substances subscripts; therefore, a score I_FLA_ + I_EXP_ + I_TOX_ = 8 is assigned. Figure 2 contributes the score of each of the sub-indices to the general index of chemical risk.

The corrosivity sub-index is the last parameter assessed parameter for CSI estimation. For this, the required construction material must be considered according to the corrosive properties of handled substances throughout the process. For the evaluated process, it was considered that stainless steel should be used in some process units given acids and alkaline substances. For this material, the assigned score is I_COR_ = 1. Equation (3) was used to calculate the chemical risk index with all the estimated chemical risk sub-indexes, obtaining a score of CSI = 16.

The second stage of the ISI method involves estimating the sub-indexes related to the process operation and structure. The PSI analysis was carried out considering that all process flows and equipment belong to the inside battery limits (ISBL) area. The plant inventory is related to the amount of material that flows through the process units considering standard residence times. The inventory was estimated as the sum of the material product flows for a residence time of 1 h. This metric is vital because the higher the mass flows in a process, the more physical stress it generates in the equipment, even though they are designed with high safety coefficients. For the evaluated process, a total inventory of 159 tons was obtained, which corresponds to a score for inventory sub-index I_I_ = 3.

The method involves estimating the risks related to the process units′ operational conditions since high pressure or temperature conditions generate physical stresses in operation, leading to accidents. In this sense, the pressure and temperature parameter scores are assigned according to these variables′ maximum quantities within the process. Table 5 shows the maximum process temperature and pressure and their corresponding score for the case of analysis. The maximum temperature (550 °C) is found in the calcination unit in the nanoparticle purification stage, while the pressure (1 atm) is the same in all the operations and equipment of the process.

The calculation of process risks requires identifying the use of process equipment such as tanks, centrifuges, dryers, mills, exchangers, evaporator, a hydrolysis reactor, and a furnace for the calcination stage. This last process equipment is significant to this indicator because the operation of these types of units is considered to be risky. The furnaces represent a score of I_EQ_ = 4, and as this value is the highest within the plant′s equipment, it is assigned for this parameter in the PSI calculation.

Finally, the safe operation structure of the process was analyzed. The evaluation of this parameter is carried out from the data reported on accidents and incidents in the operation of each type of process (for example, oil refineries, mining, biorefineries, among others). Since there are no reports of operation (and even design) of an industrial plant for the production of chitosan microbeads modified with TiO_2_ nanoparticles, this process is assigned an intermediate index, as recommended by Heikkila [29], corresponding to a score I_ST_ = 2, for neutral processes or for which there is no reported information.

**Figure 3:**
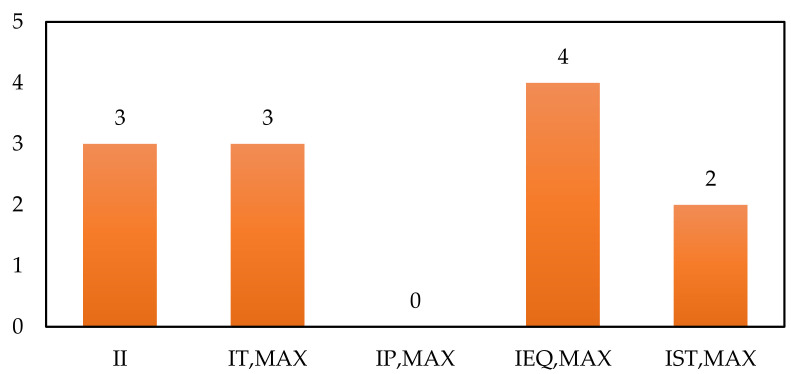
Contribution of PSI sub-indexes.

**Figure 4:**
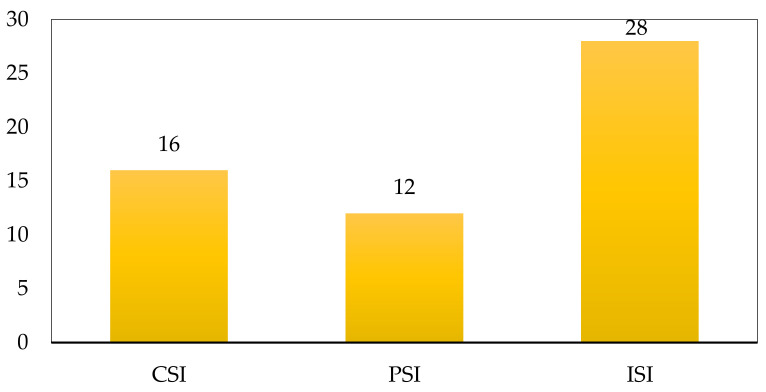
Contribution of chemical and process Inherent Safety Index (ISI) indexes.

From the estimation of the subscripts for the process safety indicator, Equation (4) was used, obtaining a general score for this parameter of PSI = 12. The global safety performance was estimated from the CSI and PSI scores, according to Equation (2). The general score obtained for the production process presented in this study was ISI = 28. Figure 3 and Figure 4 show the scores or contributions in the risk assessment for the process indicator and the inherent safety indicator, respectively.

## 4. Discussion

From a global perspective, the process obtained an ISI of 28. This result indicates that the process could present some inherent risks and hazards above the standard, which is expected to be 24 for a neutral operation. For verifying how this process would be close to operating to the recommended safety standard, this study calculated the percentage of safety operation at a neutral point (%sfn), as follows in Equation (6) [30].

(6) $$\%\mathrm{sfn}=1-\left(\frac{{\mathrm{ISI}}_{i}-{\mathrm{ISI}}_{n}}{{\mathrm{ISI}}_{n}}\right)\times 100\%$$

${\mathrm{ISI}}_{i}$ is the inherent safety index of the current evaluated process, and ISI_n_ is the inherent safety index of a neutral standard equal to 24, according to Heikkila [30]. This process obtained an %sfn=83.33%, which shows this process is close to operating at a neutral safety standard. This result might indicate that some changes can moderately reach better safety performance. Otherwise, the Chemical Inherent Safety most contributed to the resulted ISI score, with an outcome of 16. This means that this plant presents more significant inherent risks due to the substances and characteristics of the substances than the structure and equipment (and process) operation. If the CSI is observed in detail, it is evident that the high exothermic degree of the hydrolysis reaction, together with flammability and toxicity parameters of TTIP, made the CSI score to be very high for this process. Regarding the contribution of the PSI that showed a score of 12, indicating that the most significant contribution within the risk assessment is related to the furnace in the calcination process, together at the high temperatures handled in this stage. Therefore, a strategy to improve the inherent safety performance would be to find alternatives for the purification stage used in the nanoparticles synthesis stage.

## 5. Conclusions

This study presented the inherent safety analysis for chitosan microbeads modified with green TiO_2_ nanoparticles production process, using the Inherent Safety Index. Globally, the evaluated process obtained an ISI score of 28, showing a higher standard than recommended for a process considering a neutral performance. This outcome is corroborated by estimating of the percentage of safety operation at a neutral point that was equal to 83%. Results indicate the need for improvements in the process structure to reduce plant inherent risks. The safety indicator for chemical substances featured the highest score, obtaining a CSI of 16; this moderately high value is mainly related to the hydrolysis reaction performed to obtain TiO_2_. There are also risks represented by TTIP chemical nature and properties. The PSI obtained a score of 12, representing a neutral performance in terms of inherent safety, which could be improved by implementing a modification in the purification stage of the TiO_2_ nanoparticles production. Future work directions relate to process resilience metrics to complement the inherent safety analysis developed in this work. However, this approach requires more information about plant maintenance, operation, and incidents once the process is completed and under operation. Besides, evaluating techno-economic sensitivity aspects is also recommended to complement this case study and uncover improvement opportunities.

## Figures and Tables

**Table 1:** Sub-indexes for calculating Chemical Safety Index (CSI).

Dangerous Chemical Reactions Sub-Index		Reference Ranges
Heat of reaction (main)	I_RM, MAX_	0 to 4
Heat of reaction (sides)	I_RS, MAX_	0 to 4
Chemical interactions	I_INT, MAX_	0 to 4
**Dangerous Substances Sub-Indexes**		
Inflammability	I_FL_	0 to 4
Explosivity	I_EX_	0 to 4
Toxicity	I_TOX_	0 to 6
Corrosivity	I_COR_	0 to 2

**Table 2:** Sub-indexes for calculating Process Safety Index (PSI).

Process Conditions Sub-Indexes		Reference Ranges
Process inventory	I_I_	0 to 6
Process temperature	I_T, MAX_	0 to 4
Process pressure	I_P, MAX_	0 to 4
**Process System Sub-Indexes**		
Equipment	I_EQ_	0 to 4
Process structure	I_ST,MAX_	0 to 4

**Table 3:** Heat of reaction for the hydrolysis reaction.

Indicator for Main Reaction	
Main reaction	Hydrolysis
Products	
Substance	$\Delta H_{f}$ (J/g)
TiO_2_	−12,491.5
Propanol	−5292.8
Reagents	
Water	−1587.8
TTIP	−5526.5
Total heat of reaction	−24,960.92
I_RM_	4

**Table 4:** Heat of reaction for the hydrolysis reaction.

Substance	TTIP	EtOH	PrOH	AA	NaOH
Flash point (°C)	47.00	13.90	15.00	39.00	−
I_FLA_	2	3	3	2	0
UEL-LEL	−	19–3,3	2,2	−	−
I_EXP_	1	1	1	0	0
TLV (ppm)	0,86	530,71	81,36	10	1,22
I_TOX_	5	2	3	4	4
I_FLA_ + I_EXP_ + I_TOX_	8	6	7	6	4

**Table 5:** Maximum temperature and pressure indicators.

Temperature Indicator		Process Indicator	
Maximum Temperature (°C)	550	Maximum pressure (atm)	1
I_T, MAX_	3	I_P,MAX_	0

## Data Availability

The data that support the findings of this study are available in the open literature.
